# Inter-professional collaboration reduces the burden of caring for patients with mental illnesses in primary healthcare. A realist evaluation study

**DOI:** 10.1080/13814788.2019.1640209

**Published:** 2019-08-02

**Authors:** Marieke De Sutter, An De Sutter, Nora Sundahl, Tom Declercq, Peter Decat

**Affiliations:** a Department of Public Health and Primary Care, Faculty of Medicine and Health Sciences, Ghent University, Ghent, Belgium;; b Department of Radiation Oncology and Experimental Cancer Research, Ghent University Hospital, Ghent, Belgium

**Keywords:** Community mental health, staff morale, collaborative care, interprofessional relations, qualitative approach

## Abstract

**Background:** The implementation of primary care for mental health is often insufficient, which leaves its mark on staff. A team-based approach of mental healthcare prevents poor staff morale. A community health centre (CHC), therefore, set up a project promoting interprofessional collaboration with a mental health team (MHT).

**Objectives:** This study aimed to understand how an MHT would influence staff morale in a primary care setting, aiming to formulate some recommendations for future projects.

**Methods:** In 2017, interviews and a focus group discussion were conducted among the staff of a CHC. Using a qualitative approach, we aimed to unravel contextual factors and mechanisms that determine the effect of an MHT on staff morale.

**Results:** The project relieved the burden of the patient encounters and staff members felt more valuable to patients. Underlying mechanisms were recognition, altered attitudes towards patients and role clarity. Facilitating factors were intercultural care mediators and a positive team atmosphere, whereas inhibiting factors were inefficient time management and communicative issues.

**Conclusion:** Our study elucidated mechanisms and the contextual factors by which an MHT in general practice improves staff morale.

KEY MESSAGES

- An MHT improves staff morale in a CHC, through nurturing recognition, through altering staff members' attitudes and through ensuring role clarity.
- Pitfalls are inefficient time management and poor communication.
- Policymakers should stimulate interprofessional collaboration in primary mental health.

## Introduction

Primary care for mental health has many benefits, both for patients, staff and society [1–5]. Following the advice of the World Health Organization (WHO) and the World Organization of Family Doctors (Wonca) on integrating mental health into primary care, governments worldwide took action to redirect mental healthcare from institutions to community-based settings [6–8].

However, a higher number of patients with mental health illnesses increases the perceived burden and workload for primary care staff [9]. Compared to caring for patients with somatic problems, mental healthcare is more demanding in time and effort: it entails a high workload and feelings of insufficiency are more common among staff [10–18].

Given the higher influx of patients with mental health illnesses in primary care, strategies are needed to secure the wellbeing of staff and the concurrent quality of care. Although there is evidence on the positive effects of interprofessional collaboration in mental healthcare on workload, little is known about the underlying mechanisms [14,15,17,19]. Examples in literature of interprofessional collaboration comprise a more supportive work environment [5,14,15,17,19], exchange of expertise [1,3,5,20–22], and task division [5,21], though it is unknown what works, for whom, to what extent, and under which conditions. This study explored the process of an interprofessional approach by interviewing staff of a community health centre (CHC) that installed a mental health team (MHT). The results reveal contextual factors and underlying mechanisms that explain how an interprofessional collaboration in a primary care setting leads to reduced burden and improved quality in mental healthcare.

## Methods

### Setting and project characteristics

CHC Rabot, with 2200 patients registered, is located in a multicultural and socio-economically disadvantaged neighbourhood in the city of Ghent, Belgium [23]. A CHC is a multidisciplinary primary care practice in which care is financed through a capitation system in contrast to pay per performance. The staff, covering six healthcare disciplines (family medicine, physiotherapy, nursing, social work, health promotion, and psychotherapy) consisted of 25 regular employees, among which six GPs, and five volunteers.

Following the increased integration of mental health into primary care, GPs repeatedly reported on a large amount of time spent on mental healthcare and the feeling of inefficiency. To improve efficiency, the CHC installed an MHT in March 2016 (Box 1). An internal evaluation after one year showed that GPs spent less time on these patients (a decrease of 12% of GP consultations) and overall satisfaction among staff members. The present study aims to provide insight in how the implementation of interprofessional collaboration leads to this positive effect.

Box 1 Intervention

A community health centre (CHC) installed a mental health team (MHT) to make mental healthcare more efficient and less burdensome. The MHT consisted of staff members from different disciplines: psychotherapy, social work, mental health nursing, intercultural care mediation. The former two disciplines were introduced in the context of the project; the content of the latter two disciplines was expanded and/or redefined.

The MHT assisted GPs and other health professionals to share the care for patients with high psychological needs. Each of these patients was assigned to at least two health professionals with complementary expertise. The collaboration consisted of alternating patient contacts, case discussions and knowledge exchange.

To select patients for this shared care-taking, a list was compiled from the medical record systems identifying patients who consulted a GP for psychological problems more than 20 times over the last 12 months.

### Ethics

Approval was obtained from the Ethics Committee of Ghent University Hospital in May 2017 (registration number: B670201731880).

### Study design

Realist evaluation was used as a method to understand mechanisms, contextual factors and outcomes of the intervention. Realist evaluation studies have the purpose of identifying ‘what works in which circumstances and for whom?’ [24,25]. In this study, we explore how the implementation of a MHT reduces the burden of caring for patients with mental illnesses in this particular CHC, by exploring staffs’ experience. This approach allows the extrapolation of the results as it describes how this particular intervention works and what contextual factors facilitate or hinder the outcomes.

**Figure 1.**
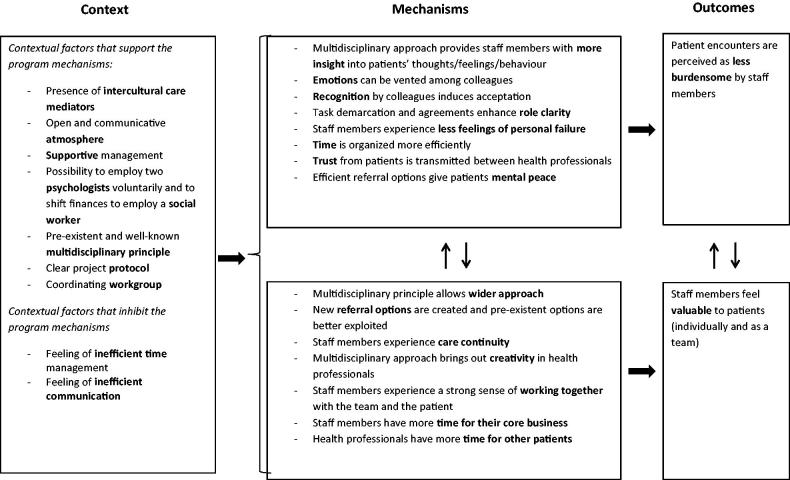
The programme mechanisms in interprofessional collaboration with a mental health team in primary care and the contextual factors that enhance and counteract these mechanisms. Programme mechanisms are the underlying mechanisms that explain how a programme works and how the outcomes are produced. The arrows denote the interaction between the factors.

Realist evaluation starts from an initial programme theory formulated in a context-mechanism outcome-structure (CMO). A grounded analysis of the study data will lead to an adapted concluding theory (Figure 1) [26]. The initial programme theory was drawn from a literature review and from staff members’ input: ‘A MHT improves staff morale in a primary care setting for the care of patients with psychological needs, through three mechanisms: (1) By being able to vent their emotions, staff members perceive the encounters with these patients as less burdensome; (2) by sharing professional knowhow, there is a personal gain for staff members; (3) by division of tasks, the workload is alleviated.’

### Recruitment of participants, data collection and data analysis

It was expected that the project implementation would affect the global CHC team. Through involving various disciplines, we intended to unravel the underlying processes in depth and from different perspectives. We selected participants among staff, including health professionals and receptionists, through a purposive sampling approach. Participants were invited face-to-face and via email. They all agreed to participate and gave informed consent. The English topic list used for the interviews is available as Supplementary material online. Data was collected through five in-depth interviews and one focus group, all taking place in the centre. Once sufficient data were collected for in-depth analysis, we stopped including new participants. From October to December 2017, five health professionals (two doctors, one nurse, one social worker and one physical therapist) were interviewed individually (each 45 min) and three receptionists were interviewed in-group (1.5 hours). The script of the interviews was based on the initial programme theory. The findings were discussed in a staff meeting of the CHC, where the interviewees were present. The feedback was used in the interpretation of the data.

The first author (MDS), GP trainee in the CHC, conducted the interviews. Field notes were taken during the interviews. The interviews were audio-recorded, de-identified and transcribed. Transcripts were imported into NVivo 11 qualitative data analysis programme (Melbourne, Australia). The first author structured the data following the CMO categories of the realist evaluation approach. This was extensively discussed with the supervisor (PD), and other authors.

## Results

### Outcomes of the intervention

Respondents in all interviews agreed upon the fact that the project was needed at the time it was introduced. All respondents expressed positive feelings towards the project and its influence on their morale.

It is not that it is impossible, but I think that—specifically for us—there are a lot of those patients on our agendas, one after the other, and that it is never easy, that it is never just a walk in the park, that it is almost always difficult. (GP)

Staff morale improvement could be divided into two outcomes. First, since the implementation of the project, patient encounters appeared to be easier to most staff members. Various mechanisms were mentioned. Some staff members described that they feel less frustrated toward a patient. Others described it more as ‘being more compassionate’, which influences patient care positively.

For me, it gives calmness. Sometimes you are frustrated because you do not achieve what you want (…) with a patient. However, sometimes you just have to accept it, that it does not always work out, and the fact that you are aware of that, makes you interact differently with the patient, which gives calmness, which in turn makes you able to focus on other things. (Social worker)

Second, staff morale appeared to be determined by the feeling of making a difference to the patient. Respondents described how the sense of ‘being stuck’ with a patient could pull down morale. When exploring this feeling, some of the respondents stated that it evolved out of not being able to offer the patient a solution. Thanks to the project, the staff members often felt that they were helping the patient better, which positively influenced their feelings.

When I know that somebody [a patient] can talk about psychological problems or lets them be treated, I feel better, because I know that the patient gets that support as well. And then it is not all carried by us, physical therapists or doctors, and somebody who had the education, is treating the patient. That makes me feel like the patient is helped better […]. It is kind of a relief, the fact that somebody else can help the patient better, psychologically or socially. (Physical therapist)

### The programme mechanisms that produce outcomes

*Patient encounters are perceived as less burdensome.* Both health professionals and other staff members mentioned that added information on patients provided by colleagues could have a substantial influence on how a staff member feels towards that patient.

The mere knowledge that other staff members also have difficulties with a certain patient was another mechanism that relieved the burden and was cited throughout all disciplines. Hearing from colleagues that they too have a hard time, that ‘it is not your fault’ and that ‘you are doing what you are able to’, had an important influence on staff members’ feelings.

But even then, it is also good to know: “Now we have thought about it with ten people, and actually, we all do not know. So it is not me, that I don’t know, we all don’t know, it is just a difficult case.” (Nurse)

Creating more role clarity was also a mechanism that came forward in several interviews.

It creates a certain peace. “Look, the doctor will join us then, we will discuss this then, now we will leave it for a moment.” […]. So for me that means a high added value and a relief. (Nurse)

Finally, trust was an important given when it came to the atmosphere of patient encounters. Throughout the project, staff members experienced that a patient’s trust towards one health professional triggered the patient also trusting their colleagues, which gave them a positive feeling.

*Staff members feel valuable to patients.* Knowing that the patient is being helped had a substantial contribution to staff members’ morale. This is not limited to the health professionals’ own efforts in the patients’ care but also applies to care given by colleagues. Respondents stated that interprofessional collaboration leads to an improved understanding of the patient, which makes it possible to approach the patients’ care more broadly, resulting in better care.

If I can express my feelings about a patient, […], that psychologist can in some way frame that patient and explain why that patient evokes this feeling in me. […]. So, even if you had the feeling of ‘being stuck’ with a patient, you can actually carry forward again with that patient, thanks to the new insights of that psychologist. (GP)

Knowing that the patient is taken care of when you are not there also eased the mind. Respondents expressed that sharing the care for a patient with a team and exchanging thoughts and feelings about that care often resulted in new ideas. These new ideas can lead to the feeling of improved caretaking. Ultimately, the project enabled health professionals to find common ground with the patient and with colleagues. This sense of teamwork is felt by health professionals and—according to the latter—by patients as well.

And I also have the feeling that those shared patients are perceived as less burdensome and that you have got fresher ideas, a fresh view […]. And I feel like it creates a feeling of “moving forward” for all parties, […], to feel like there is an evolution, that there is a growth in the cooperation, in the patient, in his functioning, whatever…. And that, for me personally, creates less burden. (Nurse)

### Contextual factors that support the programme mechanisms

The involvement of intercultural care mediators (a Turkish and a Bulgarian mediator) facilitated the process. A great deal of the patients had a Turkish or Bulgarian origin, and an important part of the psychological problems—in particular psychosomatic problems—dealt with during the project were partially influenced by or linked to cultural background. The mediators helped not only by translating but also by providing the bigger picture.

I think that the mediators to intercultural care play a vital role here, after all they have been around for a while. They know some patients very well, they know their cultural background, about which they can provide some explanation, because some people have a certain way of reasoning or thinking, they can give some tips in that matter. Often, they are a confidential adviser to patients, that is why they can be valuable when they are involved in certain conversations. (Social worker)

And it goes without saying that an open atmosphere was an important condition to enable a far-reaching collaboration, where personal boundaries can be set, where everybody feels free to speak his mind.

Among other things, we have a coordinator who can be consulted low-threshold for problems or questions […]. Also, we often meet on informal moments with the whole team to explore what creates a burden [to the team]. We also try to reinforce the team-resilience by organizing positive activities. (GP)

### Contextual factors that inhibit the program mechanisms

A recurring theme mentioned to thwart the mechanisms was inefficiency. Both inefficient time management and communicative issues came forward. Several interviewees stated that there were few official meetings, often due to personal agendas that were hard to match. When the meetings did take place, some respondents felt like there was not enough time provided for in-depth case reviews. On a communicative level, staff members stated that information was often being lost, either because it was written down in the patient file and not read, or because information was exchanged quickly in-between patients or because some staff members simply hardly saw each other.

I think it is more efficient or valuable if a certain case can be discussed in-depth. I think now we are stuffing the agenda with short five minute patient-overviews, which doesn’t really add any value […]. I believe that it would be interesting to dig out some things with which you are stuck […], until you actually know what to do with it. Instead of pronouncing after fifteen minutes: “Stop, next, time’s up!” (Doctor)

Yes, and then you have the problem that it reaches one person, but we are a large administrative team, so it also has to get to all the other people, and that is just not possible. (Receptionist)

## Discussion

### Main findings

This study shows how interprofessional collaboration can reduce the burden of caring for patients with mental health problems. Based on the analysis of the data, we adapted the initial programme theory to a concluding theory that explains how mechanisms such as recognition and role clarity contribute to this outcome (Figure 1).

### Recognition supports therapeutic presence principle

Patient encounters are perceived as less burdensome because of the possibility for staff members to vent their emotions. Support by colleagues is shown to be associated with reduced burnout [12,15]. We found that the underlying mechanism is mainly recognition. Patient encounters can be perceived as difficult because of the uncomfortable emotions caused by not being able to control the situation [19]. By sharing this feeling with colleagues, one can feel recognized and, therefore, relieved. This recognition helps health professionals to accept a sometimes-difficult situation and to realize that their mere presence and attention has a therapeutic value. In the literature, this is referred to as ‘therapeutic presence’ [27,28].

### Altered attitudes influence burden of patient encounters

Our initial programme theory comprised the hypothesis that exchanging professional know-how is one of the mechanisms explaining how the project would improve staff morale, since a lack of confidence in skills can be the reason why a patient encounter is perceived as difficult [19]. However, our findings indicate that when it comes to affecting staff morale, this information exchange is not actually about professional knowledge or skills, but rather about a change in understanding patients, resulting in altered attitudes towards the patients [11]. Patient encounters can be perceived as challenging due to certain patient behaviours, such as ‘stay sick’ or demanding behaviours [19]. Mislabelling patient behaviours, can lead to feelings of frustration, inadequacy and guilt, which contribute to the process of burnout [11]. Understanding patients’ psyche is an important strategy to cope with those problematic encounters [11,19]. On the one hand, creating new insights will make health professionals’ expectations more realistic, which promotes acceptance and supports the earlier mentioned ‘therapeutic presence’ principle. On the other hand, these new insights can trigger the health professional to try a different approach. Both can relieve the load of the patient encounters, preventing staff members from experiencing a so-called ‘compassion fatigue’.

### Role clarity contributes to improved staff morale

Team role clarity results in increased job satisfaction. Originally, we assumed that task division reduces workload, with this improving staff morale. However, our research discloses the importance of role clarity in contrast to mere task division. Role stress is experienced when a discrepancy is perceived between what staff members believe to be their role expectations and what they are capable of achieving in real practice, which is associated with higher emotional exhaustion [12]. Through a clear protocol, the project facilitates referral when needed and creates a collaboration where each fulfils its complementary role. Ensuring role clarity contributes to improving staff morale and an increased job satisfaction [14]. Hence, we recommend that the role of each discipline is clearly defined when implementing similar projects.

### Interprofessional collaboration: Threats and opportunities

As inefficiency was mentioned as a pitfall, interdisciplinary formal meetings should be organized regularly, allowing thorough case discussions and hence ensuring quantity does not beat quality.

In this study, the interprofessional collaboration focused on mental healthcare. General practice literature shows that a multidisciplinary approach also improves staff morale in the caretaking of patients with other complex needs, e.g. dementia, multimorbidity, social problems, and palliative care [29]. Consequently, the findings of this study are likely to apply to other burdensome care contexts in primary care.

### Strengths and limitations

Considering its nature, this realistic evaluation does not claim to produce universally applicable findings. However, the realistic approach has revealed mechanisms and conditions that might be suitable for similar projects aiming to decrease the burden for primary care staff attending patients with mental health problems.

The sample size was limited and not all disciplines were represented, which makes it difficult to generalize the findings to other settings. However, we did manage to involve staff from various disciplines, which adds to existing research. Research of a broader scope, involving more disciplines and using mixed methods would help us understand the mechanisms better and would improve the validity of our research.

Two of the authors, including the interviewer, are GPs at CHC Rabot. This could have influenced the given statements in the interviews as a positive evaluation could help to maintain the project.

Notwithstanding these limitations, the validity of the study was reinforced through: the long-lasting engagement in the study field as two authors are permanently working as GPs in the CHC; the feedback of the findings during a staff meeting; the gradually supervised rollout of the research; and the critical reflections of the different authors in all phases of the study.

### Policy implications

Primary care for mental health is cost-effective, and collaboration with mental health specialists is one of the strategies promoted by the WHO and Wonca [1]. This study guides us to believe that an interprofessional approach might be a way to reduce the burden of primary care teams in caring for mental health patients. Literature indicates that patients and healthcare systems benefit from mentally resilient teams [11–13,17,18]. Although further research is needed to confirm our findings, we join the current recommendations on integrating mental health into primary care, adding our suggestion that interprofessional collaboration should be taken into account when developing local and national mental health programmes.

## Conclusion

Policymakers should stimulate interprofessional collaboration in primary mental health.

The implementation of an interprofessional MHT providing shared care-taking to selected patients from a CHC improved staff morale. The intervention relieved the burden of patient encounters and staff felt more valuable to patients. Underlying mechanisms that lead to these outcomes were related to the recognition of emotions among staff members and role clarity. Intercultural care mediators and a positive team atmosphere facilitated the effect of the intervention, whereas inhibiting factors were inefficient time management and communication issues. Taken together, we believe that stimulating interprofessional collaboration can aid in the further improvement of mental healthcare in general practice.

## Supplementary Material

Topic List Interviews

Coreq Checklist
