# Supplementary material for: Inter-professional collaboration reduces the burden of caring for patients with mental illnesses in primary healthcare. A realist evaluation study
Source: Eur J Gen Pract. 2019 Aug 2;25(4):236–42. doi: 10.1080/13814788.2019.1640209 (PMC6853250; doi:10.1080/13814788.2019.1640209)
Supplement: Topic List Interviews [file IGEN_A_1640209_SM4856.docx]

**Topic list interviews**

**In-depth interview 1: Health professional 1**

| *Topic* | *Examples* |
| --- | --- |
| General | What does this project mean to you? |
| Sharing professional know-how | How does it work? |
|  | How is it organized? |
|  | What does it imply? In terms of personal and professional functioning? |
| Venting emotions | How does it work? |
|  | How is it organized? |
|  | What does it imply? In terms of personal and professional functioning? |
| Task division | How does it work? |
|  | How is it organized? |
|  | What does it imply? In terms of personal and professional functioning? |

**In-depth interview 2: Health professional 2**

| *Topic* | *Examples* |
| --- | --- |
| General | Have you experienced that the project has influenced staff morale? In what way? |
|  | What does the project mean to you? |
|  | Can you give an example? |
| Task division | How does it work? |
|  | How is it organized? |
|  | What does it imply? In terms of personal and professional functioning? For you and your colleagues? |
|  | Can you give an example? |
| Sharing professional know-how | How does it work? |
|  | How is it organized? |
|  | What does it imply? In terms of personal and professional functioning? For you and your colleagues? |
| Venting emotions | How does it work? |
|  | How is it organized? |
|  | What does it imply? In terms of personal and professional functioning? For you and your colleagues? |

**In-depth interview 3: Health professional 3**

| *Topic* | *Examples* |
| --- | --- |
| General | Have you experienced that the project has influenced staff morale? In what way? |
|  | What does it mean for you? |
|  | Can you give an example? |
| Task division | How does it work? |
|  | How is it organized? |
|  | Does the project influence time management? In what way? |
|  | What does it imply? In terms of personal and professional functioning? |
|  | Can you give an example? |
| Sharing professional know-how | How does it work? |
|  | How is it organized? |
|  | What does it imply? In terms of personal and professional functioning? |
| Venting emotions | How does it work? |
|  | How is it organized? |
|  | What does it imply? In terms of personal and professional functioning? |

**In-depth interview 4: Health professional 4**

| *Topic* | *Examples* |
| --- | --- |
| General | Have you experienced that the project has influenced staff morale? In what way? |
|  | What does it mean for you? |
|  | Can you give an example? |
| Interdisciplinary approach | How does it work? |
|  | How is it organized? |
|  | What does it imply? In terms of personal and professional functioning? For you and your colleagues? |
|  | Can you give an example? |
| Cultural aspects | Does it influence staff morale? |
|  | Is it taken into account in the project? |
|  | What does it imply? In terms of personal and professional functioning? |
| Venting emotions | How does it work? |
|  | How is it organized? |
|  | What does it imply? In terms of personal and professional functioning? |

**In-depth interview 5: Health professional 5**

| *Topic* | *Examples* |
| --- | --- |
| General | Have you experienced that the project has influenced staff morale? In what way? |
|  | What does it mean for you? |
|  | How does it work? |
| Interdisciplinary approach | How does it work? |
|  | How is it organized? |
|  | What does it imply? In terms of personal and professional functioning? |
|  | Can you give an example? |
| Cultural aspects | Does it influence staff morale? |
|  | Is it taken into account in the project? |
|  | What does it imply? In terms of personal and professional functioning? |
| Shared care-taking and creativity | How do you experience this? |
|  | What does it imply? In terms of personal and professional functioning? |
| Shared care-taking and understanding patients | How do you experience this? |
|  | What does it imply? In terms of personal and professional functioning? |
| Venting emotions | How does it work? |
|  | How is it organized? |
|  | What does it imply? In terms of personal and professional functioning? |

**Focus group discussion: Receptionists**

| *Topic* | *Examples* |
| --- | --- |
| General | Which emotions does the project evoke in you? |
| Interdisciplinary approach | How does it work? |
|  | Does the project influence task demarcation for you? |
|  | What does it imply? In terms of personal and professional functioning? |
|  | Can you give an example? |
| Shared care-taking and understanding patients | How do you experience this? |
|  | What does it imply? In terms of personal and professional functioning? |
| Venting emotions | How does it work? |
|  | How is it organized? |
|  | What does it imply? In terms of personal and professional functioning? |
