# Supplementary material for: Inter-professional collaboration reduces the burden of caring for patients with mental illnesses in primary healthcare. A realist evaluation study
Source: Eur J Gen Pract. 2019 Aug 2;25(4):236–42. doi: 10.1080/13814788.2019.1640209 (PMC6853250; doi:10.1080/13814788.2019.1640209)
Supplement: Coreq Checklist [file IGEN_A_1640209_SM4855.docx]

| **No. Item** | **Guide questions/ description** | **Reported on Page #** |
| --- | --- | --- |
| **Domain 1: Research team and reﬂexivity** |  |  |
| *Personal Characteristics* |  |  |
| 1. Interviewer/ facilitator | Which author/ s conducted the interview or focus group? | Marieke De Sutter |
| 2. Credentials | What were the researcher’s credentials? E.g. PhD, MD | MD, |
| 3. Occupation | What was their occupation at the time of the study? | General practitioner in training in the concerned community health centre. |
| 4. Gender | Was the researcher male or female? | Female |
| 5. Experience and training | What experience or training did the researcher have? | Training was provided by the supervisor (Peter Decat) in qualitative interviewing, realist evaluation and qualitative data analysis was. |
| *Relationship with participants* |  |  |
| 6. Relationship established | Was a relationship established prior to study commencement? | All participants were staff of the same community health centre of the interviewer. |
| 7. Participant knowledge of the interviewer | What did the participants know about the researcher? E.g. personal goals, reasons for doing the research. | Transparency is one of the main principles of realist evaluation. |
| 8. Interviewer characteristics | What characteristics were reported about the interviewer/ facilitator? E.g. bias, assumptions, reasons and interests in the research topic. | The fact that the interviewer is a colleague has been mentioned as possible bias in the limitations. |

| **Domain 2: study design** |  |  |
| --- | --- | --- |
| *Theoretical framework* |  |  |
| 9. Methodological orientation and theory | What methodological orientation was stated to underpin the study? E.g. grounded theory, discourse analysis, ethnography, phenomenology, content analysis | Critical realist grounded theory |
| *Participant selection* |  |  |
| 10. Sampling | How were the participants selected? E.g. purposive, convenience, consecutive, snowball | Purposive sampling |
| 11. Method of approach | How were participants approached? E.g. face-to-face, telephone, mail, email | Eight participants |
| 12. Sample size | How many participants were in the study? | Eight participants |
| 13. Non-participation | How many people refused to participate or dropped out? Reasons? | None |
| *Setting* |  |  |
| 14. Setting of data collection | Where was the data collected? E.g. home, clinic, workplace | At the workplace  . |
| 15. Presence of non-participants | Was anyone else present besides the participants and researchers? | No |
| 16. Description of sample | What are the important characteristics of the sample? E.g. demographic data, date | Divergent disciplines (2 GPs, social worker, physical therapist, nurse, 3 receptionists). |
| *Data collection* |  |  |
| 17. Interview guide | Were questions, prompts, guides provided by the authors? Was it pilot tested? | A script was provided that was discussed with the supervisor and a staff member. |
| 18. Repeat interviews | Were repeat interviews carried out? If yes, how many? | The results were presented and discussed during a staff meeting where participants were present. |
| 19. Audio/ visual recording | Did the research use audio or visual recording to collect the data? | Audio recording |
| 20. Field notes | Were ﬁeld notes made during and/ or after the interview or focus group? | yes |
| 21. Duration | What was the duration of the interviews or focus group? | Interviews lasted approximately 45 minutes; the focus group lasted 90 minutes. |
| 22. Data saturation | Was data saturation discussed? | Researchers decided to stop including new participants, once they noticed that sufficient data were collected for aanalysis in depth of the original programme theory. |
| 23. Transcripts returned | Were transcripts returned to participants for comment and/ or correction? | no |
| **Domain 3: analysis and ﬁndings** |  |  |
| *Data analysis* |  |  |
| 24. Number of data coders | How many data coders coded the data? | 2 |
| 25. Description of the coding tree | Did authors describe the coding tree? | The software NVIVO has been used for coding, which generates automatically a coding tree. A coding tree can be provided upon request. |
| 26. Derivation of themes | Were themes identiﬁed in advance or derived from the data? | The components of the CMO hypothesis have been used as overarching structuring themes. Within these themes new themes arose gradually from the data. |
| 27. Software | What software, if applicable, was used to manage the data? | Nvivo |
| 28. Participant checking | Did participants provide feedback on the ﬁndings? | During a staff meeting |
| *Reporting* |  |  |
| 29. Quotations presented | Were participant quotations presented to illustrate the themes/ ﬁndings? Was each quotation identiﬁed? E.g. participant number | Quotations are included in the text mentioning the role of the participant in the centre. |
| 30. Data and ﬁndings consistent | Was there consistency between the data presented and the ﬁndings? | Yes, there was. |
| 31. Clarity of major themes | Were major themes clearly presented in the ﬁndings? | Yes, they were. |
| 32. Clarity of minor themes | Is there a description of diverse cases or discussion of minor themes? | The figure presents all the relevant themes |
